# Clinical Presentation and Successful Management of an Infant With Down Syndrome and COVID-19 in Riyadh, Saudi Arabia

**DOI:** 10.7759/cureus.13188

**Published:** 2021-02-07

**Authors:** Ibrahim Alsahabi, Abdulaziz Alobaidi, Ayman S Alahmari, Noof Almohsen, Abdullah H Alhamoud

**Affiliations:** 1 Department of Pediatrics, Al-Imam Abdulrahman Al Faisal Hospital, Ministry of Health, Riyadh, SAU; 2 Department of Laboratory, Al-Imam Abdulrahman Al Faisal Hospital, Ministry of Health, Riyadh, SAU; 3 Department of Laboratory, Al-Imam Abdulrahman Al Faisal Hospital, First Cluster, Ministry of Health, Riyadh, SAU; 4 Medicine, King Saud Bin Abdulaziz University for Health Sciences, College of Medicine, Riyadh, SAU

**Keywords:** covid-19, pediatric, down's syndrome, congenital heart disease

## Abstract

Coronavirus is a serious, global disease. Down syndrome (DS) is characterized by immune dysregulation, has various anatomical variations, and is considered as comorbidity. These variations mean that children with DS are at risk of developing a severe case of COVID-19 if the virus is contracted. Here, we report the first case of COVID-19 in a four-month-old infant girl with DS and congenital heart disease (CHD) who was treated in Al-Imam Abdulrahman Al Faisal Hospital in the first health cluster in Riyadh, Saudi Arabia. The medical management and clinical outcome of the infant are reviewed. The infant was admitted after having received previous treatment from other hospitals, with a deteriorating condition. The patient had developed a rash, and oxygen support was required in addition to her baseline medication (furosemide and captopril). Ten days post admission, the patient’s condition improved, and she became clinically stable. She was then discharged after two consecutive negative nasopharyngeal swabs. Based on the reported case, infants with DS and CHD should be considered a high-risk patient in terms of COVID-19 infection and require close observation.

## Introduction

Coronavirus disease 2019 (COVID-19) is considered to be a serious, global disease [1]. The disease outcomes range from asymptomatic infection to serious disease and death. The severity of COVID-19 depends on factors that play an important role in inflammation processes such as immune response, excessive inflammation developing into acute respiratory distress syndrome (ARDS), and multi-organ failure [2]. Patients with pulmonary hypertension combined with minimal cardiopulmonary reserve are likely to have more serious course of COVID-19 than the general population [3,4]. Down syndrome (DS) is a genetic disease caused by a mutation in chromosome 21 (trisomy T21) and is accompanied by improper immune response and autoimmune disease. DS is considered to be the most common chromosomal abnormality [5]. People with DS have variations in the anatomical structure of the upper respiratory zone, including larger adenoids/tonsils, smaller trachea, upper way narrowing, trachea bronchus airway malacia, and glossoptosis. These variations increase the frequency of respiratory diseases and a cause of mortality among people with DS [6]. Among people of the DS patients who were admitted to an intensive care unit (ICU), 43%-78% had lower respiratory tract infections, and 50% required ventilation support [7]. Only 2% of the recorded COVID-19 cases have been in the pediatric population; however, children with comorbidities, such as congenital heart diseases, are at high risk of becoming critically ill if they contract the virus [8-10]. We address the limited data about infants with DS who contract COVID-19 by reporting the outcome of a four-month-old infant with DS and COVID-19, who was admitted to Al-Imam Abdulrahman Al Faisal Hospital in the first health cluster in Riyadh, Saudi Arabia.

## Case presentation

A four-month-old Saudi infant girl with DS and an unrepaired atrioventricular septal defect (AVSD) is presented. She is a product of a full term with an uneventful normal spontaneous vaginal delivery (NSVD), and she had a history of admission to a neonatal intensive care unit (NICU) due to respiratory distress and a cardiac congenital defect. She also had a history of multiple visits to the emergency department for fever, shortness of breath, diarrhea, vomiting, and cough. The patient was not initially screened for COVID-19.

The infant was admitted to the hospital in Riyadh, Saudi Arabia, due to mild to moderate respiratory symptoms that were managed with nebulizers; a nasopharyngeal swab was done as part of hospital protocol to screen for COVID-19 using polymerase chain reaction (PCR). The patient stabilized and was discharged on acetaminophen and an antibiotic (the parents did not remember the name of the antibiotic). Two days later, the patient’s health deteriorated; she was feeding poorly and was not tolerating the medications, for which the family sought medical attention at another hospital in the first health cluster in Riyadh. The patient’s PCR screening test was found to be positive for COVID-19, and the test was repeated for both the patient and her mother. During this visit, they were isolated, and the results came back positive for both. Following the protocols of the first health cluster in Riyadh, both were isolated and transferred to the pediatric department in Al-Imam Abdulrahman Al Faisal Hospital, which has been the isolation hospital for COVID-19 patients during the pandemic.

During this admission, the infant had a fever and diarrhea. She developed a mouth rash that was suspected to be a fungal infection, and it was treated and improved with Daktarin oral gel (miconazole). Her oxygen saturation was lower than 94%, which improved using a nasal cannula delivering 0.5 liters of oxygen. The pulmonary edema identified via chest radiography was determined to be the result of congenital heart disease (CHD) and not related to COVID-19 infection. The patient was ill and required continuous observation to prevent any further complications. Subsequent examination daily revealed that the patient’s vitals were stable on 0.5 liters of oxygen, she was not in distress, and there was no change to her level of consciousness. A cardiovascular examination revealed an audible S1+S2 and no added sounds, also found that she was hemodynamically stable, and an examination of the abdomen found it to be soft and lax with no organomegaly. However, a chest examination revealed that there were decreased breath sounds bilaterally, and while the laboratory test results showed that the complete blood count (CBC), serum hepatic enzyme, and renal hepatic enzyme were normal, the erythrocyte sedimentation rate and C-reactive protein were elevated. She was initially started on intravenous fluid for maintenance and acetaminophen as needed. She was then treated with a broad-spectrum intravenous antibiotic, cefuroxime, for five days and also received furosemide and captopril, which improved her pulmonary edema. She eventually responded dramatically to therapy, and two consecutive swabs taken one week after she was transferred were negative. The patient was reassessed and determined to be clinically stable. She was discharged after 10 days of extensive observation on her baseline medications (furosemide and captopril).

## Discussion

This case study supports the conclusion that people with DS who have multiple comorbidities are at high risk of having significant COVID-19 infection and increased mortality and morbidity rate [11-15]. In comparison with severe acute respiratory syndrome coronavirus (SARS) and Middle East respiratory syndrome (MERS-CoV) infections, COVID-19 causes aggressive deterioration of the respiratory system, which is related to stimulating of the immune system response to the virus and activating of cytokine release syndrome [16,17]. Multiple studies have shown immunologic reaction to play an important role in DS patients with severe viral infections due to their abnormal immune response to the presence of high circulating cytokines including interleukin beta (IL-β), interleukin gamma (IL-γ), and tumor necrosis factor-alpha (TNF-α) levels, with a change in cell-mediated immune function [16-18]. Alternatively, a weak anti-inflammatory response accompanied by low interleukin 6 (IL-6) and TNF-α could explain the high susceptibility of patients with DS to infections [18]. Also, people with DS have various anatomical variations in the upper respiratory tract, defects in immune regulation, and a high rate of comorbidities commonly associated with a high risk for negative outcomes from respiratory infections [19]. In the presented case, the patient developed a moderate COVID-19 infection accompanied by an oral rash. The patient was already on furosemide and captopril to treat her CHD, and an x-ray showed pulmonary edema, which suggests poor medication compliance secondary to decreased oral intake that improved with medications.

## Conclusions

This case was reported in Saudi Arabia to shed light on DS infants with COVID-19 due to limited information about the impact of COVID-19 on DS infants with comorbidities. In conclusion, DS infant patients with COVID-19 should be considered part of the high-risk population that may progress to sever disease. Preconception counseling regarding COVID-9 management and regular follow-up care by a multidisciplinary team with a pediatrician, pulmonologist, and cardiologist on board is recommended.
